# Diagnostic and Therapeutic Challenges in Intravenous Mercury Poisoning: A Case Report

**DOI:** 10.7759/cureus.64383

**Published:** 2024-07-12

**Authors:** Shivani S Bothara, Pratapsingh Parihar, Ravishankar Patil

**Affiliations:** 1 Radiodiagnosis, Jawaharlal Nehru Medical College, Datta Meghe Institute of Higher Education and Research, Wardha, IND

**Keywords:** diagnostic challenges, supportive care, multidisciplinary approach, toxicity, sickle cell anemia, intravenous mercury poisoning

## Abstract

Intravenous mercury poisoning is a rare but severe medical emergency, often resulting from accidental exposure or intentional self-harm. We present the case of a 30-year-old male with a history of sickle cell anemia who presented with high-grade fever, vomiting, giddiness, and breathlessness following intravenous mercury self-administration. Diagnostic challenges included distinguishing symptoms of acute mercury toxicity from those of his chronic condition of sickle cell trait. Markedly elevated serum mercury levels confirmed the diagnosis, with high-resolution computed tomography (HRCT) imaging studies revealing mercury deposits and alveolar lung injury. Management involved antidote of mercury poisoning, including non-invasive ventilation and transfusions, with consultations from multiple specialties. The patient demonstrated significant clinical improvement and was discharged with scheduled follow-ups. This case underscores the complexity of diagnosing and managing intravenous mercury poisoning, highlighting the importance of a comprehensive multidisciplinary approach for optimal patient outcomes.

## Introduction

One heavy metal that has been shown to have harmful effects on several organ systems is mercury. It comes in many forms, with different toxicokinetics and toxicodynamics: elemental mercury, inorganic mercury, and organic mercury compounds. Since elemental mercury can penetrate cell membranes and accumulate in tissues, it is especially dangerous when breathed in or administered intravenously. It is frequently found in instruments like thermometers and sphygmomanometers [1]. Mercury injections intravenously are extremely uncommon and are usually connected to either unintentional self-harm or unintentional exposure in medical environments [2]. Mercury toxicity can have both acute and long-term effects, and it is dose-dependent. Fever, gastrointestinal distress, respiratory compromise, and neurological abnormalities are some of the signs of acute mercury poisoning; nephrotoxicity, neurotoxicity, and dermatological effects are also observed [3].

A strong index of suspicion is necessary for the diagnosis of mercury poisoning, particularly in unusual circumstances such as intravenous injection. Elevated serum or urine mercury levels are usually the means by which laboratory confirmation is obtained. Characteristic features, such as pulmonary infiltrates or mercury deposits, may be shown by imaging techniques such as chest X-rays and high-resolution computed tomography (HRCT) [4]. Chelation therapy in extreme cases, supportive care, and an immediate stop of exposure are all part of the management of mercury poisoning. Chelation therapy's significance in elemental mercury poisoning, however, is still up for debate because of the scant evidence supporting its effectiveness and possible drawbacks [5]. In order to prevent recurrence and guarantee thorough patient care, multidisciplinary coordination is essential, especially when treating the psychological elements of purposeful poisoning [6]. This case study demonstrates the diagnostic and therapeutic challenges in managing a patient with intravenous mercury poisoning complicated by a pre-existing psychiatric condition and sickle cell anemia. The interdisciplinary approach and prompt medical intervention were pivotal in the patient's recovery, underscoring the importance of integrated care in complex toxicological emergencies.

## Case presentation

A 30-year-old male, a ward boy by occupation, presented with a history of high-grade intermittent fever, vomiting, giddiness, and breathlessness, persisting for two days. The symptoms began after the patient self-administered intravenous mercury, having broken a sphygmomanometer in the hospital. The patient reported experiencing fever without chills or rigor, multiple episodes of non-projectile vomiting containing food particles, and increasing breathlessness. There was no history of loss of consciousness or seizures. The patient's medical history revealed a known case of sickle cell anemia (hemoglobin AS pattern) for which he was not on any documented medication. Notably, he had a history of two previous suicide attempts. There was no history of hypertension, diabetes, bronchial asthma, or tuberculosis.

The patient was admitted to the intensive care unit. Upon general physical examination, the patient was in serious distress with a pulse rate of 142/min, respiratory rate of 32/min, blood pressure of 120/70 mmHg, and afebrile. Systemic examination indicated reduced bilateral air entry with bilateral basal crepitations in the respiratory system. A cardiovascular assessment revealed normal S1 and S2 heart sounds without murmurs and a conscious, oriented central nervous system. The abdominal examination was unremarkable, with no tenderness or organomegaly.

**Figure 1:**
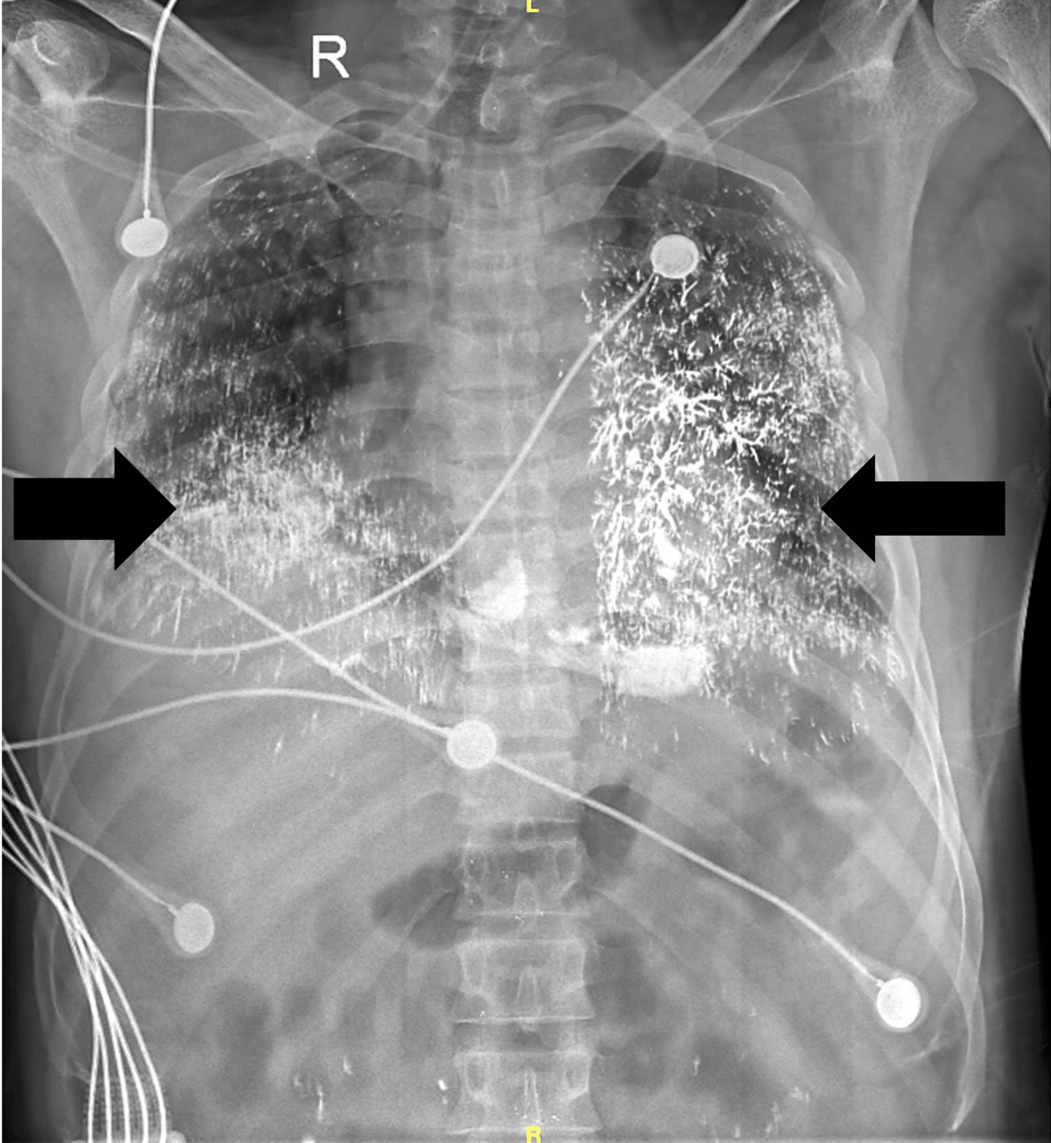
Chest radiograph shows multiple foci of rounded opacities throughout bilateral lung fields in a branching pattern (black arrows) with a history of elemental mercury poisoning.

**Figure 2:**
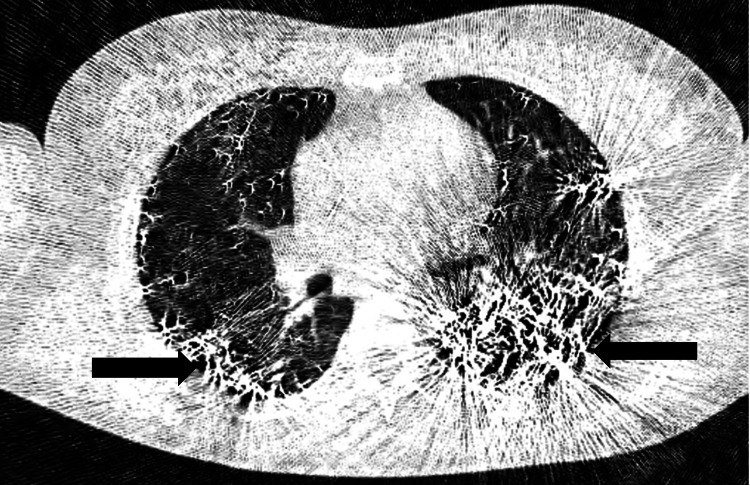
HRCT thorax (lung window ) shows scattered high attenuation foci in bilateral lung parenchyma, mostly in peripheral distribution(black arrows). HRCT - high-resolution computed tomography

**Figure 3:**
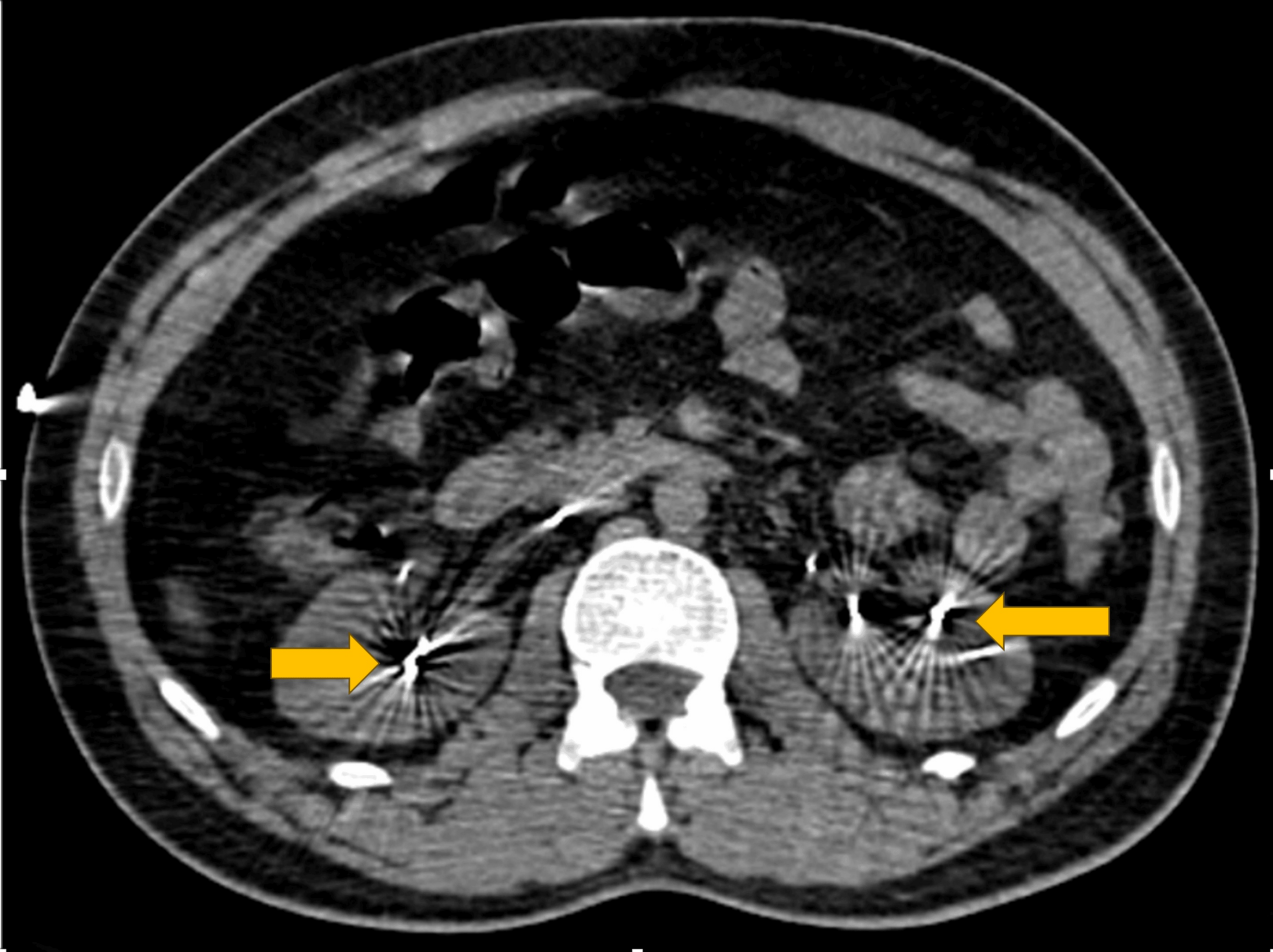
CT scan of abdomen and pelvis axial section (soft tissue window) at the level of bilateral kidneys shows foci of high attenuation in bilateral kidneys (yellow arrows), suggesting excretion of mercury through kidneys.

Laboratory investigations showed a reduced hemoglobin level of 10.6 g/dL, elevated total leukocyte count of 20,400/µL, and raised total bilirubin at 2.4 mg/dL. Critically, serum mercury levels were significantly elevated at 250 µg/dL. Chest X-ray findings (Figure 1)were consistent with acute respiratory distress syndrome (ARDS) featuring mercury embolism. An ECG indicated sinus tachycardia, while HRCT thorax (Figure 2) revealed scattered high attenuation foci in the lungs and kidneys, suggesting mercury poisoning and alveolar injury in the lungs. Computed tomography (CT) scan of the abdomen and pelvis (Figure 3) (soft tissue window) revealed foci of high attenuation in bilateral kidneys, suggesting excretion of mercury through the kidneys.

The patient received comprehensive care throughout their stay in the hospital. He was started with an injection of Dimercaprol (British anti-Lewisite (BAL)) - the antidote for mercury poisoning,150 mg deep intramuscular twice daily (BD) for 11 days. The treatment also included a transfusion of three units of packed red cells in view of reduced hemoglobin. He was managed by non-invasive ventilation with positive end-expiratory pressure (PEEP) of 6 cmH_2_O due to tachypnea and tachycardia and received antibiotics as part of the supportive care regimen. Consultations with a vitreoretinal surgeon, nephrologist, and psychiatrist were conducted to address the multi-system involvement and the psychiatric component of his condition. The fundus was normal, as per the vitreoretinal surgeon; the nephrologist recommended intravenous antibiotics. The psychiatrist recommended continuous supervision and further personality assessment to prevent future self-harm. Also, his blood counts and urine samples were monitored on a daily basis. The patient demonstrated significant clinical improvement during the course of his stay at the hospital for 25 days, stabilizing his vital signs and overall health. Consequently, he was discharged with explicit instructions and antibiotics, advised for constant supervision, scheduled follow-ups in the Psychiatry OPD for further evaluation, and a Medicine OPD review. The interdisciplinary approach and prompt medical intervention were pivotal in the patient's recovery from the severe toxic effects of intravenous mercury administration.

Patient consent

Written informed consent was obtained from the patient for publication of this case report.

## Discussion

A rare yet serious medical illness known as intravenous mercury poisoning usually results from inadvertent contact or intentional self-harm. The case study that is being given exemplifies the difficulties in diagnosing and treating this uncommon type of mercury toxicity. Numerous symptoms may indicate mercury poisoning, contingent on the type of mercury, the exposure method, and the dosage. Intravenous injection of elemental mercury can cause acute and severe systemic poisoning. This patient's symptoms, which include fever, vomiting, giddiness, and dyspnea, are suggestive of an acute systemic involvement [7]. The clinical picture was complicated by the patient's known sickle cell anemia, which made it crucial to differentiate between symptoms associated with his chronic illness and those resulting from mercury poisoning.

The markedly elevated serum mercury levels (250 µg/dL) confirmed the diagnosis. Imaging studies, including chest X-ray and HRCT thorax, revealed findings consistent with mercury deposits and alveolar lung injury, highlighting the utility of advanced imaging in diagnosing metallic mercury embolism [8,9]. Management of intravenous mercury poisoning requires a multifaceted approach. Dimercaprol (BAL) is the antidote for elemental mercury poisoning or any heavy metal poisoning, and treatment is primarily supportive. This patient required non-invasive ventilation for respiratory distress and transfusions to address anemia. Antibiotic therapy was initiated to prevent secondary infections, which are common in patients with respiratory compromise [10].

Chelation therapy, often used in mercury poisoning, was administered in this case due to the patient's stable renal function [11]. The primary therapeutic challenge was managing the ARDS, which was successfully addressed through supportive care. The involvement of multiple specialists, including a nephrologist, vitreoretinal surgeon, and psychiatrist, was crucial in managing the systemic effects of mercury and addressing the psychiatric component of self-harm. Continuous psychiatric supervision was recommended to prevent future self-harm attempts, emphasizing the importance of addressing underlying mental health issues in cases of intentional poisoning [12]. The patient showed significant improvement with stabilization of vital signs and overall health, highlighting the effectiveness of the interdisciplinary approach. Follow-up in both Psychiatry and Medicine OPDs was essential for monitoring potential delayed effects of mercury toxicity and ensuring mental health stability.

## Conclusions

The presented case of intravenous mercury poisoning highlights the diagnostic and therapeutic challenges associated with this rare form of heavy metal toxicity. Symptoms of breathlessness and tachycardia are common. Imaging techniques like X-ray, HRCT thorax, and CT of the abdomen and pelvis are crucial for diagnosis. Elevated urinary mercury levels and continuous vital monitoring are essential. Through a comprehensive multidisciplinary approach involving radiologists, nephrologists, and psychiatrists, prompt diagnosis, and supportive interventions, the patient demonstrated significant clinical improvement and stabilization of vital signs. The successful management of ARDS, coupled with continuous psychiatric supervision, underscores the importance of an interdisciplinary approach in complex poisoning cases.

## References

[1] (2024). Mercury and health. https://www.who.int/news-room/fact-sheets/detail/mercury-and-health.

[2] Rice KM, Walker EM Jr, Wu M, Gillette C, Blough ER (2014). Environmental mercury and its toxic effects. J Prev Med Public Health.

[3] Clarkson TW, Magos L (2006). The toxicology of mercury and its chemical compounds. Crit Rev Toxicol.

[4] Eastmond CJ, Holt S (1975). A case of acute mercury vapour poisoning. Postgrad Med J.

[5] Risher JF, Amler SN (2005). Mercury exposure: evaluation and intervention the inappropriate use of chelating agents in the diagnosis and treatment of putative mercury poisoning. Neurotoxicology.

[6] Rajkumar V, Lee VR, Gupta V (2024). Heavy metal toxicity. https://www.ncbi.nlm.nih.gov/books/NBK560920/.

[7] Clarkson TW, Magos L, Myers GJ (2003). The toxicology of mercury--current exposures and clinical manifestations. N Engl J Med.

[8] Park JD, Zheng W (2012). Human exposure and health effects of inorganic and elemental mercury. J Prev Med Public Health.

[9] Bjørklund G, Dadar M, Mutter J, Aaseth J (2017). The toxicology of mercury: current research and emerging trends. Environ Res.

[10] (2024). Mercury | Toxicological Profile | ATSDR. https://wwwn.cdc.gov/TSP/ToxProfiles/ToxProfiles.aspx.

[11] Kosnett MJ (2013). The role of chelation in the treatment of arsenic and mercury poisoning. J Med Toxicol.

[12] Nierenberg DW, Nordgren RE, Chang MB (1998). Delayed cerebellar disease and death after accidental exposure to dimethylmercury. N Engl J Med.

